# Teadenol B as a Component of Microorganism-Fermented Tea Extract Inhibited Breast Cancers by Promoting Autophagy

**DOI:** 10.3390/molecules29040872

**Published:** 2024-02-16

**Authors:** Ying Zhao, Zhang-Gui Ding, Yu-Jie Yan, Rui Yang, Miao-Miao Qi, Shu-Kang Pan, Ji-Ling Xie, Yu-Hui Sun, Jin Xiang

**Affiliations:** 1 Ministry of Education Laboratory of Combinatorial Biosynthesis and Drug Discovery, School of Pharmaceutical Science, Wuhan University, Wuhan 430071, China; zying1305@whu.edu.cn (Y.Z.); yanyujie524@163.com (Y.-J.Y.); qmmwhu@126.com (M.-M.Q.); yhsun@whu.edu.cn (Y.-H.S.); 2 Pu-erh Tea Fermentation Engineering Research Center of Yunnan Province, Kunming 650271, China; dzg@ynu.edu.cn (Z.-G.D.); yangrui_xyz@163.com (R.Y.); psk.happy@163.com (S.-K.P.); xiejl520@126.com (J.-L.X.); 3 Key Laboratory of Pu-erh Tea Processing Technology, Ministry of Agriculture and Rural Affairs, Kunming 650271, China; 4 Yunnan Dayi Microbial Technology Co., Ltd., Kunming 650271, China; 5 Yunnan Institute of Microbiology, School of Life Sciences, Yunnan University, Kunming 650091, China; 6 Key Laboratory for Southwest Microbial Diversity of the Ministry of Education, Yunnan University, Kunming 650091, China

**Keywords:** teadenol B, breast cancer, autophagy

## Abstract

Breast cancer is a significant threat to life and health, which needs more safe and effective drugs to be explored. Teadenol B is a characteristic chemical component of microbial fermented tea. This study discovered that teadenol B could exhibit obvious inhibitory effects on all four different clinical subtype characteristics of breast cancer cells. Proteomic studies show that deoxycytidine triphosphate deaminase (DCTD), which could block DNA synthesis and repair DNA damage, had the most significant and consistent reduction in all four types of breast cancer cells with the treatment of teadenol B. Considering MDA-MB-231 cells exhibit poor clinical prognosis and displayed substantial statistical differences in KEGG pathway enrichment analysis results, we investigated its impact on the size and growth of MDA-MB-231 triple-negative breast tumors transplanted into nude mice and demonstrated that teadenol B significantly suppressed tumor growth without affecting body weight significantly. Finally, we found that the conversion of LC3-I to LC3-II in MDA-MB-231 increased significantly with teadenol B treatment. This proved that teadenol B could be a strong autophagy promotor, which explained the down-regulation of DCTD to some extent and may be the potential mechanism underlying teadenol B’s anti-breast cancer effects. This finding provides new evidence for drinking fermented tea to prevent breast cancer and highlights the potential of teadenol B as a novel therapeutic option for breast cancer prevention and treatment, necessitating further investigations to clarify its exact target and the details involved.

## 1. Introduction

Breast cancer is the most common malignant disease in women [1], affecting more than 1 million women worldwide, with a significant increase in the number of cases worldwide, and patients tend to be younger [2]. Breast cancer cells are clinically classified according to whether they express three proteins: estrogen receptor (ER), progesterone receptor (PR), and human epidermal growth factor receptor 2 (HER2). It is usually divided into luminal A (ER/PR+/HER2−), luminal B (ER+/PR+/HER2+, or ER+/PR+/HER2− grade 3) [3,4], HER2 positive type (ER−/PR−/HER2+), and three negative type (ER−/PR−/HER2−) [5]. Among them, triple-negative breast cancer is characterized by a high mitosis rate, high lymphocytic infiltration rate, high grade, large tumor size, high recurrence rate, and high mortality rate. It is a type of breast cancer with the worst prognosis in clinical treatment at present, and it is urgent to develop new therapeutic methods [6,7]. According to different clinical tumor subtypes, it mainly includes the following three therapies: endocrine therapy, anti-HER2-targeted therapy, and chemotherapy [8,9,10]. At present, the clinical drugs for the treatment of breast cancer are easy to tolerate and have large side effects on the body, resulting in poor treatment effects [9]. Therefore, it is essential to develop safe and effective new breast cancer prevention and treatment drugs.

Fermented Pu-erh tea is a microbially fermented tea that can play a positive role in maintaining the health of the body [11,12,13] and has the effects of anti-fatigue [14,15], reducing blood lipid [16,17,18] and preventing cancers [19,20,21]. It contains many bioactive substances, mainly polyphenols, catechins, flavonoids, phenolic acids, tea pigments, saponins, polysaccharides, alkaloids, vitamins, amino acids, organic acids, and other chemical components that have been studied and reported [22]. Most of the substances are common substances in tea [23], and very few compounds have been reported to be isolated only from ripe Pu-erh tea. Thus, researching the biological activity of the characteristic chemical components in Pu-erh tea is fundamental for studying the functional mechanism of Pu-erh tea.

**Figure 1:**
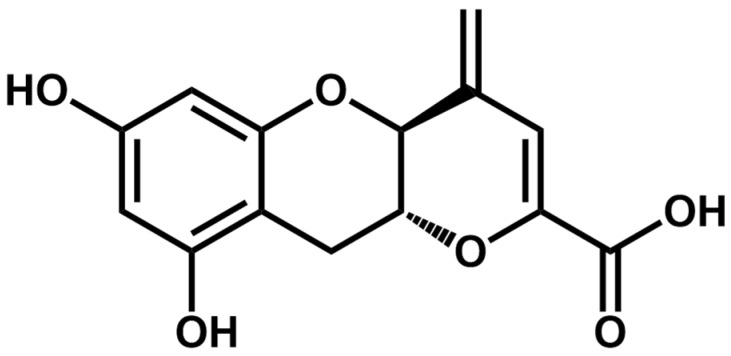
Chemical structure of teadenol B.

Teadenol B (Figure 1) is a characteristic chemical component of ripe Pu-erh tea, which was first disclosed by Japanese scholars in 2011 and isolated from the metabolites of fermented tea by Aspergillus fungi. It has been reported to promote adiponectin secretion and inhibit protein tyrosine phosphatase 1B expression [24].

Total synthesis of teadenol B was reported in 2016 [25]. It has been reported that teadenol A, which was isolated simultaneously, can bind and activate G protein-coupled receptor 120 to enhance the expression of glucagon-like peptide 1. Although related patents have been applied for teadenol B in many countries and regions [26,27,28], no other functional activities of teadenol B have been reported at present [29]. We extracted teadenol B from fermented tea and identified its chemical structure as described in our patent [28]. In the bioactivity detection of teadenol B, we first found that it showed an inhibitory effect on breast cancer cells and subsequently launched a series of cell, proteome, and animal studies to explore its role in preventing and treating breast cancer tumors and related mechanisms.

## 2. Results and Discussion

### 2.1. Teadenol B Has Inhibitory Activity on Breast Cancer Cells

**Figure 2:**
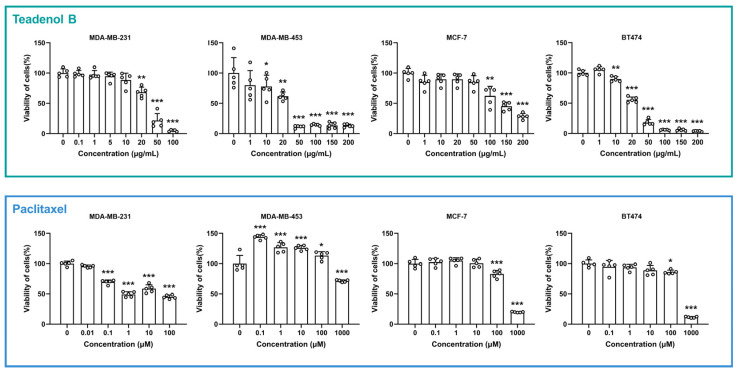
Survival rate of four types of breast cancer cells varied with teadenol B or paclitaxel concentration. The white circles represent the scatter data points for each group. The data are presented as mean ± SD (* *p* < 0.05, ** *p* < 0.01, *** *p* < 0.001, *n* = 5).

To explore whether teadenol B has therapeutic effects on breast cancer, four breast cancer cell lines were selected as research objects in this study according to receptor phenotypic characteristics, namely MDA-MB-231 (receptor phenotypic characteristics ER−, PR−, HER2−), MDA-MB-453 (receptor phenotypic characteristics ER−, PR−, HER2+), MCF-7 (receptor phenotypic characteristics ER+, PR+/−, HER2−), and BT474 (receptor phenotypic characteristics ER+, PR+/−, HER2+). Breast cancer cells were treated with a range of concentrations of teadenol B for 24 h, and the cell viability was detected by adding an MTT solution. As shown in Figure 2, MDA-MB-231 cell viability was significantly inhibited by teadenol B (*p* < 0.01) after its concentration reached 20 μg/mL. For MDA-MB-453 cells, the cell survival rate decreased when the concentration of teadenol B reached 10 μg/mL (*p* < 0.05). The cell survival rate decreased significantly (*p* < 0.001) after the concentration of teadenol B reached 50 μg/mL. When teadenol B concentration reached 100 μg/mL, the survival rate of MCF-7 cells decreased significantly (*p* < 0.01). As to BT474 cells, the survival rate decreased significantly after the concentration of teadenol B reached 10 μg/mL (*p* < 0.01).

Paclitaxel, a chemotherapeutic drug for breast cancers, was used as a positive control drug here. As shown in Figure 2, after the concentration of paclitaxel reached 0.1 μM, the survival rate of MDA-MB-231 cells significantly decreased (*p* < 0.001). Paclitaxel promoted the proliferative activity of MDA-MB-453 cells in the concentration range not exceeding 100 μM. The survival rates of MCF-7 cells and BT474 cells were much higher than 50% even when the concentration of paclitaxel reached 100 μM.

By comparing the half-maximal inhibitory concentration (IC50) of teadenol B and paclitaxel in these four cell types (Table 1), we can see that the IC50 of teadenol B is much lower in MDA-MB-453 and BT474 cells. For MCF-7 cells, the IC50 of teadenol B is roughly at the same level as that of paclitaxel. For MDA-MB-231 cells, the IC50 of teadenol B is higher than paclitaxel, but from the morphology of the dose-response curve, the inhibition of teadenol B increased with concentration more clearly and regularly, which can also be seen in the results of the other three types of breast cancer cells.

### 2.2. Proteomic Study of the Potential Mechanism of Action of Teadenol B against Breast Cancer

Teadenol B can inhibit the growth of breast cancer cells. To further explore the potential mechanism of its anti-breast cancer action, breast cancer cells, MDA-MB-231, MDA-MB-453, MCF-7, and BT474 were treated with 20 µg/mL teadenol B and their proteins were extracted and treated. The proteins of the teadenol B-treated group were compared with those of the control group using label-free MS. The summary data of all the differentially expressed proteins are shown in Table 2.

**Figure 3:**
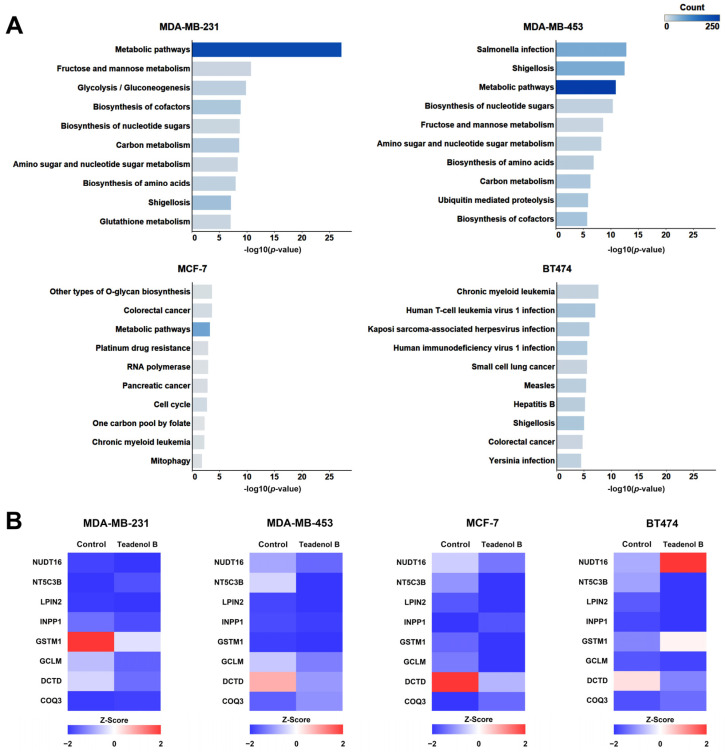
Teadenol B inhibits cancer cell growth by affecting key proteins on metabolic pathways in breast cancer cells. (**A**) KEGG pathway enrichment analysis of teadenol B intervention on differential protein expression in four breast cancer cells. The *p*-value is the scoring value of Fisher’s exact test. The smaller the *p*-value is, the larger the value of the horizontal axis -log10 (*p*-value), which means that the difference between the sample group and the control group in the corresponding longitudinal KEGG pathway is more significant. The count represents the number of differentially expressed proteins on the KEGG pathway. (**B**) The heat map showed differences in the expression of common proteins in the metabolic pathways, and teadenol B had a notable effect on the four breast cancer cells.

The Kyoto Encyclopedia of Gene and Genome (KEGG) access database (www.kegg.jp/kegg/pathway.html, accessed on 17 January 2024) stores the function of gene and genome information, along with visual representations of various cellular biochemical processes. Analyzing the metabolic pathways enriched with differentially expressed proteins allows for an understanding of which pathways experience notable systemic changes under differing experimental conditions. In this study, KEGG pathway analysis was performed on proteins with significant expression differences compared with the control group to discover the biological pathways by which teadenol B interferes with four types of breast cancer cells. Figure 3A lists the enrichment analysis results of the KEGG pathway with the top 10 protein expression differences according to Fisher’s precise test.

As shown in Figure 3A, the protein with significant differences in metabolic pathways (hsa01100) was the most significant, and the number of different proteins was the most significant after teadenol B interfered with MDA-MB-231 cells. In MDA-MB-453 cells, the protein with significant differences in expression was also mainly enriched in metabolic pathways (hsa01100), with the highest number of differentially expressed proteins. The difference was also significant, second only to Salmonella infection pathways (hsa05132) and Shigellosis pathways (hsa05131). Teadenol B interferes with MCF-7 cells, and the significantly differentially expressed protein is also mainly enriched in metabolic pathways (hsa01100). The significance of the difference is second only to other types of O-glycan biosynthesis pathways (hsa00514) and colorectal cancer pathways (hsa05210), but the difference is the weakest compared with the other three types of breast cancer cells. Teadenol B interferes with BT474 cells, and the most significant difference is between the chronic myeloid leukemia pathway (hsa05220) and the human T-cell leukemia virus (hsa05166), and the number of the different proteins in these two pathways is not very different from its pathway.

After teadenol B treatment, the four types of cells had multiple proteins with the same changes in metabolic pathways (Figure 3B). The expression of lipin 2 (LPIN2), glutamate-cysteine ligase modifier subunit (GCLM), and deoxycytidine triphosphate deaminase (DCTD) decreased. The expression of coenzyme Q3 (COQ3) increased. Among them, DCTD is closely related to anticancer effects, which shows the most significant changes in all four types of breast cancer cells with the treatment of teadenol B.

### 2.3. Teadenol B Inhibits the Growth of Transplanted Tumors in Nude Mice

Triple-negative breast cancer is characterized by a high mitosis rate, high lymphocytic infiltration rate, high grade, large tumor size, high recurrence rate, and high mortality rate. It is a type of breast cancer with the worst prognosis in clinical treatment at present, and it is urgent to develop new therapeutic methods. KEGG pathway analysis results (Figure 3A) showed that among the four different breast cancer cells above, MDA-MB-231 cells had the most significant difference in protein expression and the largest number of different proteins produced by the intervention of teadenol B. Therefore, triple-negative breast cancer cell MDA-MB-231 was selected as the animal study object to further explore the effect of teadenol B on the growth status, size, and toxicity of transplanted tumors in nude mice.

**Figure 4:**
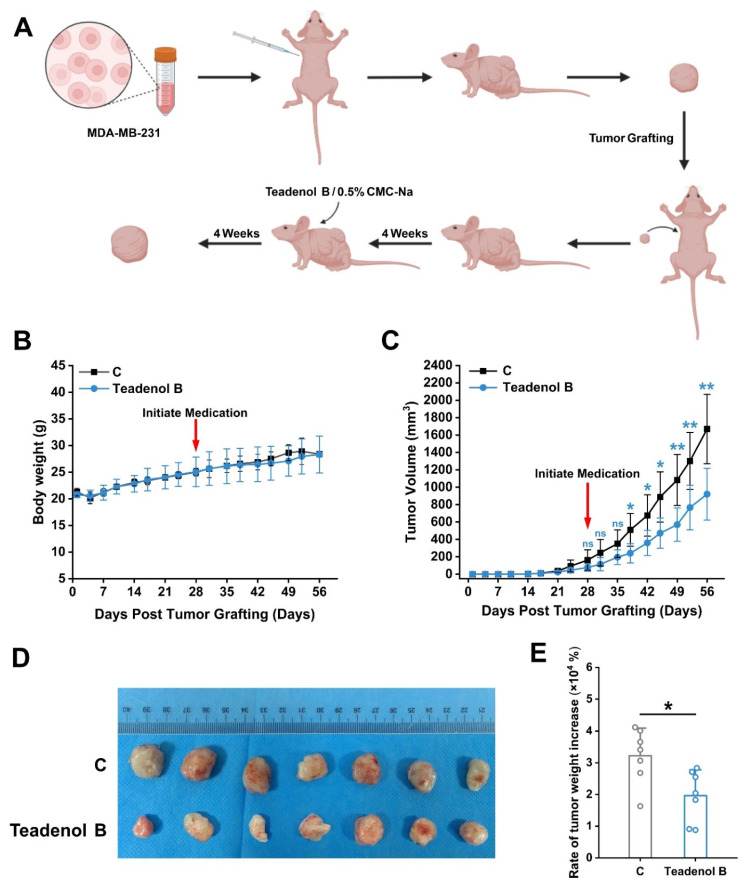
Inhibitory effect of teadenol B on the transplanted tumor in nude mice. (**A**) Flowchart of tumor transplantation and drug treatment in nude mice; (**B**) changes in body weight of nude mice over time. The data are presented as mean ± SD (*p* > 0.05, *n* = 7); (**C**) the changes in breast tumor volume with time in nude mice were statistically significant from 2 to 4 weeks after administration. The data are presented as mean ± SD (ns, *p* > 0.05; * *p* < 0.05, ** *p* < 0.01, *n* = 7); (**D**) at week 8, mice were sacrificed for extract untreated and teadenol B-treated tumor tissues; (**E**) the effect of administration of teadenol B on the rate of breast tumor weight increase was statistically significant after four weeks of administration. The data are presented as mean ± SD (* *p* < 0.05, *n* = 7).

Cultured MDA-MB-231 breast cancer cells were injected into the axillary region of 2 nude mice with a prepared cell suspension (containing approximately 5 × 10^6^ cells) in a volume of 0.1 mL. Once the tumors reached a certain size, they were dissected into small fragments and transplanted into an additional 20 nude mice. Four weeks after tumor transplantation, the model group received intragastric administration of 0.5% CMC-Na at a dose of 10 mL/kg, while the treatment group was administered teadenol B at a dose of 75 mg/kg every three days for four weeks (Figure 4A). Results demonstrated that breast tumor volume significantly decreased after two weeks of treatment (Figure 4B), indicating effective inhibition of breast tumor growth in nude mice by gastric administration of teadenol B. Moreover, the rate of increase in breast tumor weight was significantly reduced after four weeks of treatment (Figure 4C), confirming excellent anti-breast cancer activity exhibited by teadenol B. Furthermore, no statistically significant difference was observed in body weight changes between the control group receiving an equivalent volume of solvent via oral gavage and the group treated with teadenol B (Figure 4D). These findings preliminarily suggest that this dosage and route do not induce significant toxic reactions.

### 2.4. Teadenol B Inhibits MDA-MB-231 Cells by Inhibiting DCTD as an Autophagy Promotor

**Figure 5:**
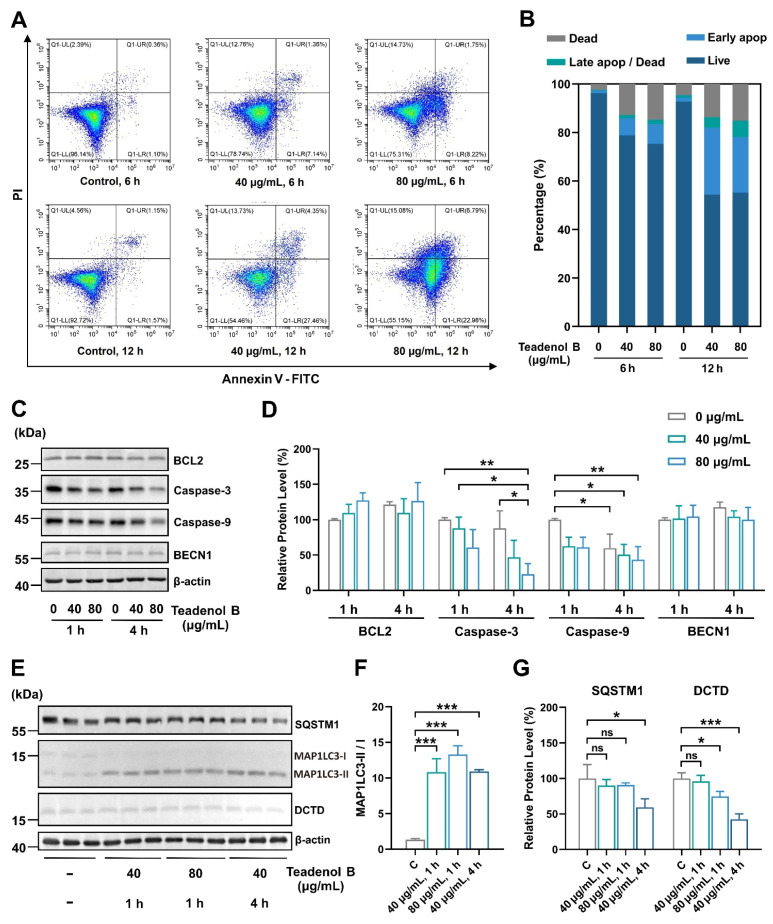
Teadenol B can promote autophagy and reduce the expression level of DCTD to inhibit the growth of MDA-MB-231 cells. (**A**) Teadenol B promotes apoptosis in MDA-MB-231 cells; (**B**) teadenol B mainly promotes early apoptosis in MDA-MB-231 cells; (**C**) changes in the expression levels of apoptosis-related proteins in MDA-MB-231 cells after treatment with teadenol B; (**D**) the semi-quantitative analysis. The data are presented as mean ± SD (* *p* < 0.05, ** *p* < 0.01, *n* = 3); (**E**) changes in the expression levels of autophagy-related proteins and anti-cancer-related proteins in MDA-MB-231 cells after treatment with teadenol B; (**F**,**G**) the semi-quantitative analysis. The data are presented as mean ± SD (ns, *p* > 0.05; * *p* < 0.05, *** *p* < 0.001, *n* = 3).

According to the proteomic results (Figure 3B), the DCTD protein may be the key protein involved in the inhibition of teadenol B on cancer cell growth. Considering the close relationship between DCTD and apoptosis, we conducted further experiments and found that after treatment with teadenol B, both early and late apoptosis of MDA-MB-231 cells increased, with a more significant increase in early apoptosis. This indicates that teadenol B primarily inhibits cancer cell growth by inducing programmed cell death in MDA-MB-231 cells while maintaining the integrity of the cell membrane (Figure 5A,B). One hour after treatment with teadenol B, there was a slight increase in the expression level of BCL2, but with no statistical difference (*p* > 0.05). We also examined the levels of the uncut forms of caspase-9 and caspase-3 (Figure 5C,D). Compared to the control group treated for 1 h, the expression levels of the uncut form of caspase-9 decreased significantly after 4 h of treatment with teadenol B (40 µg/mL, *p* < 0.05; 80 µg/mL, *p* < 0.01). Compared to the control group treated for the same duration, 80 µg/mL of teadenol B significantly reduced the protein level of the uncut form of caspase-3 (*p* < 0.05). This further demonstrates that teadenol B can induce apoptosis in cells.

As shown in Figure S1, the proteomic results show that teadenol B changes the expression of some proteins in the autophagy pathway. ATG12, ATG3, BCL2, and RELA expression decreased, and mTOR was most significantly down-regulated (Figure S2). Thus, we hypothesized that teadenol B might play a role in promoting autophagy. We examined some autophagy proteins, including the most widely used markers (Figure 5E–G). String analysis results show that these proteins interact with the apoptotic proteins mentioned above (Figure S3). After 4 h of treatment with 40 µg/mL of teadenol B, the protein level of SQSTM1 in MDA-MB-231 cells decreased significantly (*p* < 0.05) with a significant increase in the MAP1LC3-II/I ratio (*p* < 0.001), which represents a high degree of conversion from MAP1LC3-I to MAP1LC3-II. Additionally, teadenol B significantly reduced the protein level of DCTD (*p* < 0.001), and this effect was shown to be enhanced with increasing concentration or treatment time of teadenol B. All these results proved that teadenol B was an autophagy promotor, which is consistent with the results of proteomics in Figure S2. The protein level of DCTD decreases (Figure 5E,G), which might be due to the intracellular protein degradation role of autophagy.

### 2.5. Discussion

Breast cancer is a serious threat to women’s lives and health, among which triple-negative breast cancer is a kind of breast cancer with the worst prognosis in clinical treatment. Ripe Pu-erh tea and microbial fermented tea have positive effects on maintaining body health and preventing cancers [20,21,22]. Identification of the biological activity of the characteristic chemical components of Pu-erh tea is the basis for the study of the functional mechanism of Pu-erh tea. Teadenol B is a common compound in Pu-erh ripe tea and microbial fermented tea. Except for the known adiponectin secretion-promoting activity and protein tyrosine phosphatase 1B expression inhibitory activity, no other activities have been reported [24]. In this study, in the screening process of anti-breast cancer activity using the MTT test, it was found that teadenol B showed inhibitory effects on four types of breast cancer cells corresponding to different clinical subtypes, including MDA-MB-231 triple-negative breast cancer cells. Among them, the inhibitory effect on MDA-MB-231, MDA-MB-453, and BT474 breast cancer cells was stronger, and the inhibitory effect on MCF-7 breast cancer cells was slightly weaker. Furthermore, proteomic studies were conducted to investigate the potential mechanism of action of teadenol B against breast cancer, and it was found that metabolic pathways (hsa01100) were the most significantly different cell signaling pathways under the intervention of teadenol B, which provided information basis for subsequent studies on its anticancer mechanism. Considering that MDA-MB-231 cells had the worst clinical prognosis and showed the most significant statistical difference in the KEGG pathway enrichment analysis results, to further clarify the inhibitory effect of teadenol B on breast cancer, the interventional effect of teadenol B on the size and growth of MDA-MB-231 triple-negative breast cancer grafts in nude mouse animal models was studied. The experimental results showed that teadenol B significantly inhibited the growth of breast cancer in nude mice but had no significant effect on the body weight of nude mice, indicating that teadenol B could inhibit MDA-MB-231 triple-negative breast cancer grafts. There was no obvious toxic reaction.

Through proteomics studies, we found that the metabolic pathway is the most affected by teadenol B. Comparing the results of the detected protein changes in the metabolic pathways, we found that only DCTD’s changes were consistent in all four cell types, with significant downregulation. It is speculated that the change in this protein may be one of the reasons for the inhibition of all four types of cancer cells. It has been reported that the degradation of DCTD could block DNA synthesis and the repair of DNA damage [30]. DNA damage can stimulate BH3-only proteins to bind with high affinity to anti-apoptotic BCL2 proteins, activating pro-apoptotic effectors and triggering a cascade of caspase reactions, leading to orderly cell apoptosis [31]. Cell apoptosis is one of the inherent mechanisms in the cell cycle that can promptly eliminate non-functional, harmful, and abnormal cells [32].

Autophagy is one of the important pathways for intracellular protein degradation, and it is closely related to the occurrence and development of cancer. In simple terms, the deficiency of autophagy in cells can lead to the accumulation of misfolded proteins, which can induce carcinogenesis; excessive autophagy can also induce cell apoptosis. Therefore, theoretically, autophagy promotors have potential preventive and therapeutic effects against cancer [33]. Some autophagy promotors, such as rapamycin [34], resveratrol [35], metformin [36], and carnosic acid [37], have been reported to possess anticancer activities. Here, we found that the conversion of MAP1LC3-I to MAP1LC3-II increased extremely significantly with the treatment of teadenol B, which proved teadenol B to be an autophagy promotor that can be used to explain the down-regulation of DCTD to some extent.

## 3. Materials and Methods

### 3.1. Cell Culture

All experiments used cells within 20 passages. Both MDA-MB-231 (CELL-C0150, Bioswamp, Wuhan, China) and MDA-MB-453 (CELL-C0152, Bioswamp, Wuhan, China) were cultured in L-15 medium with phenol red (Jinuo, Hangzhou, China) supplemented with 10% fetal bovine serum (FBS, 11011-8611, Sijiqing, Hangzhou, China) in a 37 °C incubator. MCF-7 (CC0302, Cellcook, Guangzhou, China) were cultured in MEM medium with phenol red (MA0217, Meilunbio, Dalian, China) supplemented with 10% FBS and 10 µg/mL insulin (118975, Aladdin, Shanghai, China) at 37 °C in a 5% CO_2_ atmosphere. BT474 (CC0310, Cellcook, Guangzhou, China) were cultured in RPMI 1640 medium with phenol red (MA0215, Meilunbio, Dalian, China) supplemented with 10% FBS and 10 µg/mL insulin at 37 °C in a 5% CO_2_ atmosphere. The media were replaced with phenol red-free RPMI 1640 medium (11835, TBD, Tianjin, China) before all the experiments were conducted to avoid estrogenic stimulation of the phenol red.

### 3.2. MTT Assays

The impact of teadenol B on cell viability and proliferation was assessed using MTT assays. Cells (8 × 10^4^ cells/mL) were plated in 96-well plates and incubated overnight at 37 °C with 5% CO_2_. The culture medium was replaced with 100 μL phenol red-free and serum-free RPMI-1640 medium, and then the cells were exposed to paclitaxel (P106869, Aladdin, Shanghai, China) or teadenol B, extracted and identified as described in our patent [28], alone for 24 h. The 1H, 13C NMR data of teadenol B are presented in Table S1. The purity of teadenol B is greater than 95%. After washing once with phosphate buffer saline (PBS, MA0015, Meilunbio, Dalian, China), 100 µL of MTT solution (0.5 mg/mL, Kerui, Wuhan, China) was added to each well, and the cells were incubated at 37 °C with 5% CO_2_ for 4 h. After aspirating the culture medium, 150 µL dimethylsulfoxide (DMSO, 30072418, SCR, Shanghai, China) was added to each well. The optical density (absorbance) at 570 nm was measured with a microplate reader (Spark, Tecan, Switzerland). Cell viability was determined by calculating the percentage of viable cells in teadenol B-treated samples compared to vehicle control-treated cells, which were considered 100% viable. Each experiment was independently repeated five times.

### 3.3. Proteomic Sample Preparation

MDA-MB-231 cells were plated into 6-well plates at a density of 5 × 10^5^ cells/mL and incubated at 37 °C with 5% CO_2_. After 24 h, the culture medium was replaced with 2 mL phenol red-free and serum-free RPMI-1640 medium, and then the cells were exposed to PEG400 (0.1%) and teadenol B (20 µg/mL) alone for 1 h. After culture, the cells were washed twice with PBS (4 °C) and scraped off the culture dish into a centrifuge tube using a cell scraper. The cell suspension was centrifuged at 1000× g and 4 °C for 1 min, after which the supernatant was removed. Finally, the cells were stored at −80 °C for later testing and analysis.

### 3.4. Label-Free MS Analysis

Serum-starved cells were exposed to a treatment of 0.1% PEG400 and 20 µg/mL teadenol B for 1 h to explore the effects of teadenol B on breast cancer cells. After washing with cold PBS, the cells were collected and subjected to dialysis, concentration, and quantification using the BCA method. The protein samples were adjusted to an equal concentration, dried, trypsin-digested, and then analyzed using mass spectrometry (Thermo Fisher, Orbitrap Fusion, Waltham, MA, USA). The resulting mass spectrometry raw files were searched against the SwissProt protein database using MaxQuant software (version 1.6.5.0) with a false discovery rate (FDR) ≤ 1%. The IBAQ (intensity-based absolute protein quantification) algorithm was employed for quantitative analysis. A comparison of the teadenol B-treated group with the control group was performed to identify proteins with similar differences. Additionally, a pathway analysis based on KEGG was conducted.

### 3.5. Human Breast Cancer Xenograft Nude Mice Model

Male BALB/c nude mice were purchased from Wuhan Bestcell Model Bio-Tech Co., Ltd. (Wuhan, China). MDA-MB-231 cells were re-suspended in medium and mixed with Matrigel matrix (356231, Corning, New York, NY, USA), resulting in a final cell concentration of 5 × 10^7^ cells/mL, and injected into the armpits of 5 to 6-week-old male nude mice. After the tumors reached approximately 1 cm^3^, they were cut into 10 mm^3^ pieces and then transplanted into 20 male BALB/c nude mice aged 5 to 6 weeks and weighing 20 to 22 g, respectively. Mice were randomly divided into control and teadenol B groups (10 mice per group). Four weeks after the tumor transplant, oral administration was started. Teadenol B was dissolved in 0.5% sodium carboxymethyl cellulose (CMC-Na, M9317, AbMole, Houston, TX, USA). Teadenol B was given by oral intake at a dose of 75 mg/kg/3d. The administration period was 4 weeks. Tumors were measured at seven-day intervals with a caliper, and tumor volume was calculated using the following formula: volume (mm^3^) = width^2^ × length × 6/π. At the end of the administration cycle, Tumor tissue was taken and weighed to calculate the rate of breast tumor weight increase. All the animal studies obeyed the principles of the 1983 Declaration of Helsinki. They were conducted following the Chinese Regulations for the Administration of Affairs Concerning Experimental Animals and performed in accordance with the Guidelines of the China Animal Welfare Legislation. This study was approved by the Hubei Provincial Science & Technology Department, Wuhan, China (Approval Number: SYXK 2022-0124).

### 3.6. Western Blot

MDA-MB-231 cells were exposed to 0.1% PEG400 and teadenol B (40, 80 µg/mL) for 1 and 4 h to assess the influence of teadenol B on the expression levels of proteins associated with autophagy. Following treatment, the harvested cells were directly lysed in 200 µL of ice-cold lysis buffer containing protease inhibitors. The resulting lysates were then subjected to centrifugation at 12,000× *g* at 4 °C for 10 min. Subsequently, a loading buffer was added at a 1:1 ratio, and the samples were collected and separated on SDS-polyacrylamide gels. The proteins were then transferred to nitrocellulose filter membranes and washed in Tris-buffered saline (TBS) with 0.1% Tween20 (TBST) 3 times. The membranes were incubated overnight with antibodies for BECN1 (1:10,000, 66665-1-Ig, Proteintech, Chicago, USA), caspase-3 (1:500, A19654, Abclonal, Wuhan, China), caspase-9 (1:500, 10380-1-AP, Proteintech, Chicago, IL, USA), DCTD (1:1000, 68357-1-Ig, Proteintech, Chicago, USA), MAP1LC3 (1:1000 dilution, 14600-1-AP, Proteintech, Chicago, IL, USA), and SQSTM1 (1:2000 dilution, 66184-1-Ig, Proteintech, Chicago, IL, USA), respectively, at 4 °C overnight. The membranes were then washed with TBST and incubated with HRP-conjugated Affinipure Goat Anti-Mouse IgG(H+L) (1:2000 dilution, SA00001-1, Proteintech, Chicago, IL, USA) at 37 °C for 2 h. Finally, the proteins were visualized using an Enhanced HRP-DAB Chromogenic Substrate Kit (4994100, Tiangen, Beijing, China). The antibody β-actin (1:10,000 dilution, 66009-1-Ig, Proteintech, Chicago, IL, USA) was used as the loading control. Each experiment was repeated in triplicate. The quantification of the blots was performed using Photoshop CC 2018.

### 3.7. Apoptosis Assay

Apoptotic activity was assessed using the Annexin V-FITC/PI staining kit (P-CA-201, Procell, Wuhan, China), containing Annexin V-FITC, propidium iodide (PI), and Annexin V Binding Buffer. MDA-MB-231 cells were plated at a density of 5 × 10^5^ cells per well in 6-well plates. Following treatment with the drug, the cells underwent two PBS washes, followed by harvesting and centrifugation at 300× *g* for 5 min. Subsequently, the cells were exposed to 500 μL of 1×Annexin V-FITC Buffer along with 5 μL of Annexin-V and 5 μL of PI for 20 min at room temperature in the absence of light. Annexin staining was quantified using a flow cytometer (CytoFLEX S, Beckman, CA, USA).

### 3.8. Statistical Analysis

The results are reported as the mean ± SD. The statistical analysis of the data was performed using one-way or two-way ANOVA, followed by Tukey’s post hoc test as applicable, to assess for any differences. A *p*-value greater than 0.05 indicated no statistically significant difference. Statistical significance was defined as *p* < 0.05, indicating a significant finding. *P* < 0.01 was considered highly significant, while *p* < 0.001 was considered extremely significant.

## 4. Conclusions

In summary, this study proved the inhibitory effect of teadenol B on the viability of four breast cancer cells, including MDA-MB-231 breast cancer. It preliminarily revealed that its anticancer activity may be related to its inhibitory effect on DCTD as an autophagy promotor, but further research is needed to clarify the details involved in the future. In addition, different types of breast cancer cells have different sensitivity to teadenol B, so the therapeutic value of teadenol B for different breast tumors needs further research. This work provides a new basis for the healthcare efficacy of drinking microbially fermented tea in the prevention and treatment of breast cancer, expands the research scope of the active mechanism and application of teadenol B compound, and also provides a new choice for the development of new drugs for the prevention and treatment of breast cancer.

## Figures and Tables

**Table 1:** IC50 of teadenol B and paclitaxel in four types of breast cancer cells.

Cell Line	Receptor Phenotypic Characteristics	Teadenol B		Paclitaxel	
		(µg/mL)	(µM)	(µg/mL)	(µM)
MDA-MB-231	ER−, PR−, HER2−	35	126	64	75
MDA-MB-453	ER−, PR−, HER2+	30	108	>850	>1000
MCF-7	ER+, PR+/−, HER2−	145	525	425	500
BT474	ER+, PR+/−, HER2+	25	90	425	500

**Table 2:** Statistics of the number of differentially expressed proteins.

Cell Line	Up Regulation (fc ≥ 2)	Down Regulation (fc ≤ 0.5)	Differentially Expressed Proteins
MDA-MB-231	473	872	1345
MDA-MB-453	472	1604	2076
MCF-7	272	416	688
BT474	331	932	1263

## Data Availability

Data are contained within the article and Supplementary Materials.

## Supplementary Materials

The following supporting information can be downloaded at: https://www.mdpi.com/article/10.3390/molecules29040872/s1, Figure S1: Differentially expressed proteins related to autophagy pathways in MDA MB 231 cells; Figure S2: Heat map analysis of differentially expressed proteins related to autophagy pathways in MDA MB 231 cells; Figure S3: Interaction analysis of key proteins using STRING database; Table S1: ^1^H, ^13^C NMR data for teadenol B (solvent: deuterated dimethyl sulfoxide (DMSO-d_6_)).

## References

[1] Kolak A., Kaminska M., Sygit K., Budny A., Surdyka D., Kukielka-Budny B., Burdan F. (2017). Primary and secondary prevention of breast cancer. Ann. Agric. Environ. Med..

[2] McGuire A., Brown J.A., Malone C., McLaughlin R., Kerin M.J. (2015). Effects of age on the detection and management of breast cancer. Cancers.

[3] Sviderskiy V.O., Blumenberg L., Gorodetsky E., Karakousi T.R., Hirsh N., Alvarez S.W., Terzi E.M., Kaparos E., Whiten G.C., Ssebyala S. (2020). Hyperactive CDK2 Activity in Basal-like Breast Cancer Imposes a Genome Integrity Liability that Can Be Exploited by Targeting DNA Polymerase ε. Mol. Cell.

[4] Zhang Y., Tian J., Qu C., Peng Y., Lei J., Sun L., Zong B., Liu S. (2020). A look into the link between centrosome amplification and breast cancer. Biomed. Pharmacother..

[5] Zhao L., Han X., Lu J., McEachern D., Wang S. (2020). A highly potent PROTAC androgen receptor (AR) degrader ARD-61 effectively inhibits AR-positive breast cancer cell growth in vitro and tumor growth in vivo. Neoplasia.

[6] Qi M., Liu X., Zhou Y., Wang H., Zhao Y., Ren J., Xiang J. (2021). Berberine Inhibits MDA-MB-231 Cells as an Agonist of G Protein-Coupled Estrogen Receptor 1. Int. J. Mol. Sci..

[7] Xiang J., Wang Y., Su K., Liu M., Hu P.C., Ma T., Li J.X., Wei L., Zheng Z., Yang F. (2014). Ritonavir binds to and downregulates estrogen receptors: Molecular mechanism of promoting early atherosclerosis. Exp. Cell Res..

[8] Harbeck N., Gnant M. (2017). Breast cancer. Lancet.

[9] Barzaman K., Karami J., Zarei Z., Hosseinzadeh A., Kazemi M.H., Moradi-Kalbolandi S., Safari E., Farahmand L. (2020). Breast cancer: Biology, biomarkers, and treatments. Int. Immunopharmacol..

[10] Maughan K.L., Lutterbie M.A., Ham P.S. (2010). Treatment of breast cancer. Am. Fam. Physician.

[11] Brody H. (2019). Tea. Nature.

[12] Yang C.S., Wang Z.Y. (1993). Tea and cancer. J. Natl. Cancer Inst..

[13] Trevisanato S.I., Kim Y.I. (2000). Tea and health. Nutr. Rev..

[14] Camfield D.A., Stough C., Farrimond J., Scholey A.B. (2014). Acute effects of tea constituents L-theanine, caffeine, and epigallocatechin gallate on cognitive function and mood: A systematic review and meta-analysis. Nutr. Rev..

[15] van Dam R.M., Hu F.B., Willett W.C. (2020). Coffee, Caffeine, and Health. N. Engl. J. Med..

[16] Sirotkin A.V., Kolesárová A. (2021). The anti-obesity and health-promoting effects of tea and coffee. Physiol. Res..

[17] Jeukendrup A.E., Randell R. (2011). Fat burners: Nutrition supplements that increase fat metabolism. Obes. Rev..

[18] Vázquez Cisneros L.C., López-Uriarte P., López-Espinoza A., Navarro Meza M., Espinoza-Gallardo A.C., Guzmán Aburto M.B. (2017). Effects of green tea and its epigallocatechin (EGCG) content on body weight and fat mass in humans: A systematic review. Nutr. Hosp..

[19] Sinha D., Biswas J., Nabavi S.M., Bishayee A. (2017). Tea phytochemicals for breast cancer prevention and intervention: From bench to bedside and beyond. Semin. Cancer Biol..

[20] Mao X., Xiao X., Chen D., Yu B., He J. (2019). Tea and Its Components Prevent Cancer: A Review of the Redox-Related Mechanism. Int. J. Mol. Sci..

[21] Zhao L.G., Li Z.Y., Feng G.S., Ji X.W., Tan Y.T., Li H.L., Gunter M.J., Xiang Y.B. (2021). Tea Drinking and Risk of Cancer Incidence: A Meta-Analysis of Prospective Cohort Studies and Evidence Evaluation. Adv. Nutr..

[22] Zhu M.Z., Li N., Zhou F., Ouyang J., Lu D.M., Xu W., Li J., Lin H.Y., Zhang Z., Xiao J.B. (2020). Microbial bioconversion of the chemical components in dark tea. Food Chem..

[23] Lv S., Wu Y., Zhou J., Lian M., Li C., Xu Y., Liu S., Wang C., Meng Q. (2014). The study of fingerprint characteristics of Dayi Pu-Erh tea using a fully automatic HS-SPME/GC-MS and combined chemometrics method. PLoS ONE.

[24] Wulandari R.A., Amano M., Yanagita T., Tanaka T., Kouno I., Kawamura D., Ishimaru K. (2011). New phenolic compounds from *Camellia sinensis* L. leaves fermented with *Aspergillus* sp.. J. Nat. Med..

[25] Song J.-H., Miyazaki H., Yoshida S. (2017). Simple Method for the Preparation of Teadenols A and B by a Combined Process of Submerged Culture with *Aspergillus* sp. and Chromatographic Separation. Food Sci. Technol. Res..

[26] Yanagita T., Ishimaru K., Tanaka T., Koba K., Miyazaki H., Aoki N., Kawamura D. (2015). Functional Microbially Fermented Tea Extract Containing Polyphenol Derivative and Method for Producing the Same. U.S. Patent.

[27] Yanagita T., Ishimaru K., Tanaka T., Koba K., Miyazaki H., Aoki N., Kawamura D. (2013). Polyphenol Derivative and Method for Producing the Same. U.S. Patent.

[28] Liu J., Ding Z., Gao L., Chen D., Liu M., Tang S. (2020). Methods for Separating Teadenol A and Teadenol B from Fermented Tea. CN Patent.

[29] Nagasawa T., Ishimaru K., Higashiyama S., Hama Y., Mitsutake S. (2020). Teadenol A in microbial fermented tea acts as a novel ligand on GPR120 to increase GLP-1 secretion. Food Funct..

[30] Weiner K.X.B., Ciesla J., Jaffe A.B., Ketring R., Maley F., Maley G.F. (1995). Chromosomal Location and Structural Organization of the Human Deoxycytidylate Deaminase Gene. J. Biol. Chem..

[31] Radha G., Raghavan S.C. (2017). BCL2: A promising cancer therapeutic target. Biochim. Biophys. Acta Rev. Cancer.

[32] Carneiro B.A., El-Deiry W.S. (2020). Targeting apoptosis in cancer therapy. Nat. Rev. Clin. Oncol..

[33] Xiang J., Liu X., Ren J., Chen K., Wang H.L., Miao Y.Y., Qi M.M. (2019). How does estrogen work on autophagy?. Autophagy.

[34] Sun L., Yan Y., Lv H., Li J.L., Wang Z.Y., Wang K., Wang L., Li Y.X., Jiang H., Zhang Y.Y. (2022). Rapamycin targets STAT3 and impacts c-Myc to suppress tumor growth. Cell Chem. Biol..

[35] Abdel-Latif G.A., Al-Abd A.M., Tadros M.G., Al-Abbasi F.A., Khalifa A.E., Abdel-Naim A.B. (2015). The chemomodulatory effects of resveratrol and didox on herceptin cytotoxicity in breast cancer cell lines. Sci. Rep..

[36] Yang J.J., Zhou Y.L., Xie S.D., Wang J., Li Z.Q., Chen L.N., Mao M.S., Chen C., Huang A.H., Chen Y.X. (2021). Metformin induces Ferroptosis by inhibiting UFMylation of SLC7A11 in breast cancer. J. Exp. Clin. Cancer Res..

[37] Yesil-Celiktas O., Sevimli C., Bedir E., Vardar-Sukan F. (2010). Inhibitory Effects of Rosemary Extracts, Carnosic Acid and Rosma rinic Acid on the Growth of Various Human Cancer Cell Lines. Plant Food Hum. Nutr..

