# Supplementary material for: Teadenol B as a Component of Microorganism-Fermented Tea Extract Inhibited Breast Cancers by Promoting Autophagy
Source: Molecules. 2024 Feb 16;29(4):872. doi: 10.3390/molecules29040872 (PMC10892666; doi:10.3390/molecules29040872)
Supplement: Supplementary file 1 [file molecules-29-00872-s001.zip › molecules-2854821-supplementary.pdf]

Table S1. ^{1}H, ^{13}C NMR data for teadenol B (solvent: deuterated dimethyl sulfoxide (DMSO-d_6)).

position	δ_H (mult., J_{HH} Hz) (500 MHz)	δ_C (mult.) (125 MHz)	ROESY **H↔H**	HMBC H→C
2	4.00 (1H, *d*, *J*=13.5)	71.10 (*d*)	4-H	C-3,4,14,15
3	4.42 (1H, *m*)	72.77 (*d*)	4-H	C-2,C-14
4	2.56 (1H, *m*, *J*=8.0, *J*=19.4)	25.70 (*d*)		C-2,3,5,9,10
	3.04 (1H, *m*, *J*=12.8, *J*=19.4)			C-2,3,5,9,10
5		156.38 (*s*)		
6	5.96 (1H, *d*, *J*=2.6)	95.96 (*d*)	5-OH	C-5,7,8,10
7		156.96 (*s*)		
8	5.79 (1H, *d*, *J*=2.6)	94.18 (*d*)	7-OH	C-6,7,9,10
9		154.28 (*s*)		
10		98.04 (*s*)		
12		144.00 (*s*)		
13	6.57 (1H, *s*)	111.49 (*d*)	15-H	C-2,12,14,15,16
14		136.71 (*s*)		
15	5.39 (2H *br*, *s*)	112.55 (*t*)	13-H	C-2,C-13,C-14
16		163.05 (*s*) (*s*)		
5-OH	9.46 (1H, *br*)		6-H	C-5,6,10
7-OH	9.14 (1H, *br*)		8-H	C-6,7,8

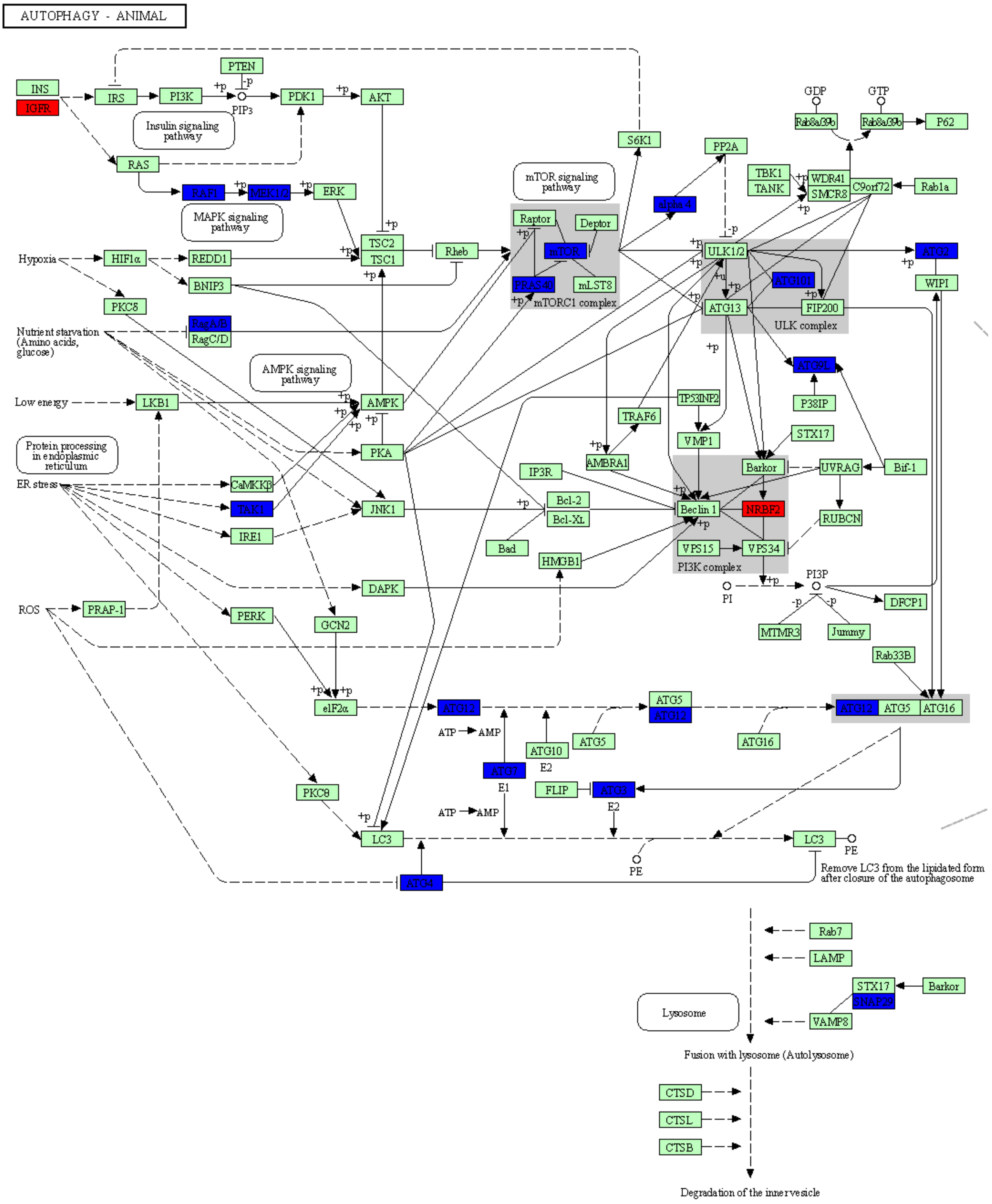

Figure S1. Differentially expressed proteins related to autophagy pathways in MDA-MB-231 cells.

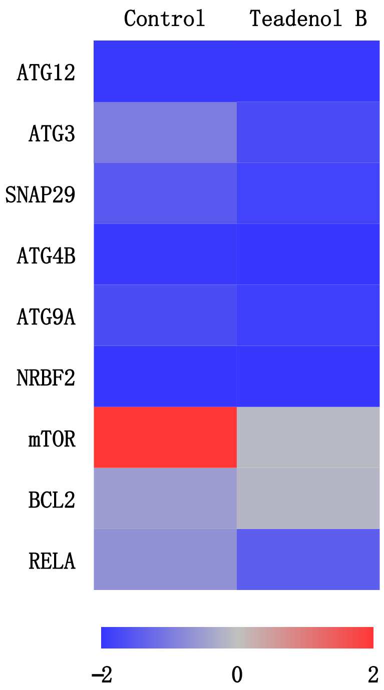

Figure S2. Heat map analysis of differentially expressed proteins related to autophagy pathways in MDA-MB-231 cells.

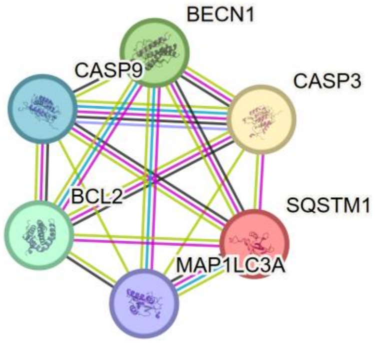

Figure S3. Interaction analysis of key proteins using STRING database.
